# Foreign

**Published:** 1841-02-20

**Authors:** 


### FOREIGN.

*Penetrating wound, severing the right anterior chord of the spinal marrow, followed by instantaneous loss of motion in the right side of the body, without loss of sensation.* By M. Begin.—Louis Antoine Lafontaine, æt. fifty-nine, strongly built, and of sanguine temperament, received a penetrating wound, *Oct. 21st*, 1840, at the posterior part of the neck. Being struck from behind, he fell instantly, and could not rise; he conceived the wound to be inflicted by a heavy instrument, to which a sharp point had been adapted, and imagined that his fall was attributable not to the puncture, but to the contusion. The fall occurred backwards and upon the right side, as was attested by the excoriation and ecchymosis. No apparent injury was exhibited in any other part of the body, although the effects were attributed by the individual himself to contusions received by the fall.

Lafontaine, an old soldier, retained his senses after the injury, and observed the results with considerable sagacity; transported to his quarters, he entertained no suspicion of the gravity of his situation; and the wound in the neck being closed by adhesive strips, he refused to be bled.

Oct. 22d. In the evening he complained of no pain, but experienced a sensation of numbness in the right side; the pulse was moderately excited; the edges of the wound, which was apparently superficial, were already united; warm cataplasms were directed to the feet, with cold applications to the head and neck, and lemonade for drink.

23d. After a calm night, a more complete examination showed that the transverse wound was perfectly united, and was apparently produced by a double-edged instrument; it was situated opposite the fifth cervical vertebra, and was accompanied neither by ecchymosis, tumefaction, heat, or pain. The motions of the neck were free, and no inconvenience was felt when they were carried to an extreme, or accompanied by considerable pressure.

The patient, however, complains of weight in the right arm, and formication in the corresponding hand; he can nevertheless raise the arm, although with difficulty, and move the forearm; but the fingers in a state of semiflexion cannot be extended, and can be only imperfectly closed; the right inferior extremity is incapable of any movement, and an obscure pain is experienced throughout the right side of the chest. The *sensibility* of these parts is perfectly normal; the face is flushed; the pulse full, but not frequent; the head rather heavy, but the intellectual and visceral functions unaltered.

There existed a singular contradiction between the apparent simplicity of this wound and the paralysis of the limbs of the corresponding side.

The patient evidently fell, not as he supposed, from the force of the blow, but from the instantaneous loss of muscular power in the right lower extremity.

The cessation of *motion alone* on the side of the wound indicated a lesion of the right anterior chord of the cervical portion of the medulla spinalis; the less perfect paralysis of the upper extremity was attributed to the fact that as the wound was situated opposite the fifth vertebra,—a portion of the brachial plexus originated above it, and this portion continued to act; and finally, if respiration remained unimpeded on the right side, it was because the roots of the phrenic nerve had escaped injury.

The prognosis was consequently regarded as very grave, although instances of cure in analogous cases are upon record.

V. S. fifteen leeches to head repeated three times during the day and night, followed by cold applications; revulsives to feet.

24th. The great toe of the right side appears capable of slight motion; the pulse is full; the skin hot; the head heavy; the tongue coated, with a disposition to dryness; continual thirst. There is a slight alteration of the countenance, which appears more emaciated; constipation; the urine passes naturally.

Venesection; forty-five leeches to the forehead and right side of the neck during the twenty-four hours; enema of oil.

25th. After a calm night, during which he slept several hours, the patient feels better; the head is lighter, and the pulse less strong and frequent; the tongue is cleaner, and the thirst moderate; the enema produced two large evacuations; the motions of the head and neck are easy, and free from pain, and the patient is desirous to rise while his bed is arranged.

With the exception of venesection, the same prescriptions are continued, with a laxative enema; notwithstanding these, fever, with cerebral congestion, agitation, and delirium, exhibit themselves during the night, and yield to the application of twenty leeches.

26th. Skin still hot; pulse frequent, but not very full; the head is burning; the temporal arteries beat forcibly; ten leeches were applied to the temple and the mastoid region of the right side; the same prescriptions continued; at 12 o'clock a violent chill supervened, followed at 2 o'clock by an incomplete reaction, which diminished and disappeared during the evening. The patient took a few teaspoonfuls of a solution of sulphate of quinine. During the night the agitation increased, a dull delirium manifested itself, and the pulse became irregular; the respiration up to this period perfectly free and normal, became more rapid and embarrassed; hiccough supervened at intervals; blisters, dusted over with camphor, were applied to the thighs, but did not act; the symptoms grew worse; the dyspnœa increased, and death occurred the 27th, at 8 A. M.

The most striking features of this observation are the isolated paralysis of motion, with entire preservation of sensation in the extremities of the right side—the absence of all pain and of all impediment to the motions of the head and neck, and the persistence of all the excretions up to the last moment.

*Autopsy.*—1. The external trace of the wound is scarcely perceptible, the cicatrix is so exact; beneath the skin a slight ecchymosis is found in the cellular tissue, which is thick and abundantly supplied with fat; in proportion as the muscular layers are raised to a greater depth, the solution of continuity becomes more apparent, and is surrounded by greater infiltration of blood; at the same time the muscular fibres are more irregularly torn; no large vessel has been opened.

2. On exposing the spinal column, the fragment of a knife blade was found on a level with the bony bridge of the sixth vertebra; the fragment projected posteriorly about two millimetres, and its back was directed towards the median line; the cervical portion of the spinal column was carefully removed; and upon detaching the soft parts from its anterior face, in order to facilitate the action of the saw, the point of the knife was discovered; this projected three millimetres between the sixth and seventh vertebræ, having broken the upper edge of the body of the latter; the point had also injured the posterior parietes of the pharynx, without entirely traversing it.

3. In order to examine more accurately the state of the spinal marrow, a section was made along the spinous processes, and another in front along the bodies of the vertebræ, to the left of the median line, so as to leave the foreign body in its situation, the bony portions of the tube being separated, and the meninges opened, it was easy to detach the medulla and extract it.

Pus was found mixed with the liquid which surrounded it, and its surface was softened above, and particularly below the wound; it had been injured by the back of the knife blade, and the section extended obliquely from the origin of the posterior roots of the spinal nerves to the anterior median fissure; while the right anterior chord of the medulla was cut from the point indicated as deep as the gutter occupied by the spinal artery; a line drawn from one of these points to the other will manifestly separate, on the outside and in front, the anterior portion of the medulla spinalis, from the inner and posterior portion which was almost if not entirely uninjured.

The fragment of the instrument which remained in the rachis, belonged to a solid and perfectly sharpened dirk knife.

The general direction of the wound was therefore oblique, from above downwards, and from without inwards, since commencing opposite the fifth vertebra on the right side, it terminated opposite the upper surface of the seventh vertebra, and to the left of the median line.

The shock which must have accompanied a penetrating wound of sufficient violence to perforate the bridge of the sixth vertebra and the intervertebral cartilage, as well as a portion of the body of the seventh vertebra, explains perfectly the sensation of contusion experienced by the patient at the moment of the injury.

4. Besides the lesions just described, nothing remarkable was discovered in the other organs; a very thin layer of blood existed beneath the arachnoid which covered the lobes of the cerebellum, and the cerebellum itself was rather more injected than the cerebrum; it is worthy of remark in connection with this appearance, that neither during life, nor after death, was any erection of the penis observed.

MM. Ollivier, (of Angers,) Gerardin, and Diess, found the lungs highly coloured in every part, and that their section permitted the escape of a large quantity of blood; the left cavities of the heart were empty, while the right were full, and contained a dark-coloured coagulum; the other viscera offered no appreciable alteration.

---

*A Clinical Lecture delivered to the Pupils of the Bristol Infirmary.** By Dr. Prichard.—Among the patients now under treatment in the wards of the Infirmary, there are several whose cases are worthy of particular attention.

I wish to draw your notice, in the first place, to a case of dropsy with albuminous urine, which has terminated in complete recovery. Michael Ryan, in Ward No. 5, was admitted on the 22d of June, labouring under ascites, with great distension of the scrotum and œdema of the legs, in a state of great debility and exhaustion. He complained also of rheumatic pains in his legs. His disorder had been brought on by exposure to cold. He had been working with his legs in mud and cold water in cleaning a dock. He had no discoverable disease of the thoracic organs, or of the liver. His urine was stated to be not very scanty, and of the natural colour. When subjected to heat, it was found to contain about two-thirds of coagulable matter: this was repeatedly done, and uniformly with the same result. Such was the permanent state of the urine for some time. Michael Ryan is now convalescent; he feels himself perfectly well; his aspect is altogether changed; he has no longer ascites or anasarca; not even œdema of the legs. His urine is perfectly natural; it has been of late repeatedly subjected to heat, without displaying albumen. He will be discharged "Recovered" in a few days. Before I advert to the practice followed in his case, I shall make a few general observations on his complaint.

The kidneys, in a state of health, separate daily from the circulating fluid so great a portion, that any considerable change either in the quantity or the quality of the urine, if it subsists for some time, must obviously exert a great influence on the physical state of the body, and on the health of the individual affected. If the quantity alone is materially diminished, some marked effects must soon follow. When the diminution is gradual, the overloaded vessels relieve themselves for a time by pouring out a part of their contents into the great cavities, and into the cellular tissue, and the patient becomes dropsical. If the morbid influence which affects the functions of the kidneys is such as to produce a sudden and entire cessation, other results ensue. In some instances a partial relief is obtained by a copious fluid secretion into the stomach and intestines, and the patient vomits great quantities of a fluid which in its sensible properties more or less resembles urine. A well-known medical practitioner, who lived some years in this city, was thus affected. He had been in indifferent health for some time, when, after exposure to cold and fatigue, he was suddenly seized with total suppression of urine. This was speedily followed by almost constant vomiting, and he threw up, during two or three days, in large quantities, a fluid which in its sensible properties bore a considerable resemblance to urine. When this vomiting ceased, which it did rather suddenly, he became affected with stupor, and died comatose. A girl who was some years since, during many months, in the Infirmary, laboured under suppression, which was not always complete, for the catheter occasionally brought off a very small quantity of urine. Every evening she threw up a large quantity of fluid, resembling urine. The fluid ejected in these cases has been seldom examined with care. In the instance to which I have last adverted it was accurately analysed, and was found to contain urea, and occasionally benzoic acid, which are well-known products of the fluid secreted by the kidneys. It was suggested to me as probable that this female practised deception, and swallowed, in the first instance, the fluid which she was seen to eject from her stomach, and that it was really her urine. She was carefully watched while in the ward (No. 4,) and I think she could not possibly have found opportunities for carrying on deception of this

* The following pages contain the substance of a clinical lecture delivered to the pupils of the Bristol Infirmary. As it explains some methods of practice which are there pursued, and which have been misrepresented, Dr. Prichard is desirous that it should appear in print, through the medium of the Medical Gazette.

kind; and had it been so, and the stomach in its usual state, the fluid would have been sometimes mixed with the ordinary contents of that organ. In another case in this Infirmary, I understand that urea and benzoic acid have been discovered in the fluid ejected under similar circumstances from the stomach. In future the greatest care will be taken to investigate similar cases.

Changes in the quality of the urine are more likely to escape notice than striking alterations in its quantity; in fact, they often exist for a long time without being discovered. We have seen cases in which the two greatest deviations known from the healthy condition of the urine have existed, without any material change in its quantity or appearance; and this indicates the necessity of an accurate examination of the urine, and of subjecting it more frequently than is usually done to chemical agents. Cases of albuminous urine are well known to have occurred without any considerable change in the natural quantity of this fluid, and without any symptoms of dropsy. I have lately attended a private patient, who, without any material alteration in the quantity of his urine, laboured under what is termed diabetes mellitus. To term one of these diseases dropsy, and the other diabetes, is an abuse of words. The characteristics of dropsy are wanting in the one case, and the character of diabetes (which is an increased flow of urine) in the other. I shall term one of these affections *leucomaturia*, and the other *melituria*. In the case of melituria to which I have alluded, the patient was affected with slowly-coming-on and gradually increasing debility, inertia, anorexia with respect to all the physical appetites; latterly of impaired vision and memory. He was evidently becoming imbecile. His urine was stated to be natural in quantity and in appearance. A constantly dry skin, with thirst, and a slight gleet, gave suspicion of the nature of his disease; and his urine, on being subjected to heat, was found to contain a large quantity of saccharine matter. Leucomaturia, as I have said, often occurs without dropsical symptoms. This disease was first clearly distinguished by Dr. Blackall, about thirty years ago, who pointed out the fact that it arises from different causes from those of ordinary dropsy, and requires a different treatment; but it was to Dr. Bright, one of the most distinguished pathological anatomists of the present age, that we are indebted for the important observation, that leucomaturia is connected with organic disease of the kidneys. It is not, however, always an incurable disease, as the present case proves. The most appropriate method of treatment is perhaps not yet ascertained. Dr. Osborne, of Dublin, who has devoted his attention to this disease, on which he is acknowledged to have thrown much light, proposes to treat it principally by exciting diaphoresis, and by that method he appears to have been very successful. I have tried his plan, but have not been able to obtain the same result. It will be right to bear in mind his observation; and if the dry state of the skin can be overcome, which is not always the case, some good result may be found to arise from the use of diaphoretics; but as the disease is often of an inflammatory nature—which we may infer from the exciting causes, and from the appearance often displayed by the blood when drawn—it is wrong to attempt its cure by stimulating sudorifics. It is only by collecting the results of experience—we may as well say at once, of experiments—on the success of remedies in the treatment of this disease that we can hope to arrive at any certain conclusions as to the method of cure; and in this point of view it will be worth while to note the means employed in the present case, of which the result has been more than ordinarily successful. I shall first observe that they are precisely the means which are generally most efficacious in the treatment of inflammatory diseases, whether of membranes or parenchymatous structures. The patient was bled twice during the first three days after his admission, and well purged with the black cathartic mixture of infusion of senna and Eposm salt, of which a dose was ordered for him three times in a day. His gums were then made sore by small doses of calomel, which were discontinued. He has from that time had no other medicine than a saline aperient mixture, containing sulphate of magnesia, with some spirit of nitric ether, and a few drops of tincture of squill. Under this treatment his progress towards recovery has been constant and uniform.

There are two patients now in the Infirmary convalescent of acute laryngitis, whose symptoms and progress you have had an opportunity of observing. One of them is George Morris, in No. 6, aged 25 years, admitted on the 25th of June; and the other, Phœbe Powells, aged 20 years, admitted July 23. In the case of Morris, acute laryngitis had supervened on a chronic disease of the trachea, which had previously lasted eight months, and which still remains after the acute disorder has been removed. The female had laryngitis complicated with extensive inflammation of the bronchial membrane: a muco-purulent rhonchus was perceptible in the respiratory action over a great part of the thorax. Both of these cases were characterised by the usual symptoms of laryngitis; a frequent shrill sharply-sounding cough, hoarseness almost amounting to loss of voice, oppressed difficult respiration, with a distressing sense of constriction about the larynx, and of breathing through a narrow and insufficient opening, which in reality is the case, since in these instances the epiglottis and its ligaments, and the whole larynx, are found inflamed and thickened, so as to lessen materially the rima glottidis, which is besides obstructed with tough mucus.

The method of treatment pursued in both these cases were similar. It consisted principally in the abstraction of blood and the use of calomel, and these are the only remedial means which are of any importance in this disease. In both instances, being used early and perseveringly, they were speedily efficacious, although the symptoms were at first severe. Morris was bled from the arm, and the female patient had leeches frequently applied to her throat. Local bleeding is generally thought the most appropriate remedy, but when there is much arterial excitement, or general inflammatory action, it is almost useless to attempt reducing the local inflammation till that state is relieved by venesection. Even in the ordinary gastro-enteritis, which is the form of continued fever that generally occurs in this hospital, we often find that one bleeding of twelve or fourteen ounces will save the application of a great many leeches to the epigastrium, and relieve the patient much more speedily and effectually. Next to bleeding the most important remedy in laryngitis is mercury. I generally order three grains of calomel with one of Dover's powder to be taken every third or fourth hour until the gums become sore, and then to be immediately discontinued. In both of these cases the disorder, which had been but partially relieved by bleeding, gave way at once as soon as the mouth became affected; the cough nearly ceased, the constriction of the throat and sense of difficult breathing approaching to suffocation was removed, and a free expectoration followed. Tartar emetic was also given to these patients in small doses, and Phœbe Powells took one dose of three grains, which produced vomiting and some temporary relief, but it was from mercury that she derived the only permanent benefit.

*Cure of Chorea.*

Charles Haynes, a little boy, aged nine years, came into the hospital on June 25th, and he has just been dismissed. You have all seen him in No. 5, labouring under chorea. When he was admitted, a cathartic draught, the ordinary purgative mixture of infusion of senna, with Epsom salt, was ordered for him, to be taken every morning, and this, with the addition of the shower bath, was all the medical treatment that he underwent. He immediately began to improve, and left the Infirmary, as you know, quite well. Chorea is a disease of the brain, that is, the pathological state which is the immediate cause of the symptoms, is an affection of some part of the encephalon. This does not appear obvious from the ordinary phenomena of the complaint, which consist nearly in jactitation of the limbs: but the inference may be drawn, first, from the connection of chorea with other confessedly cerebral diseases, such as epilepsy, with which it is often combined; secondly, from the consequences of long-continued chorea, which are similar to those of epilepsy and paralysis, namely, dementia, or an obliteration of the mental faculties: it is not uncommon to see children half idiotic from chorea, and they become in time completely so, if the disease is not arrested; thirdly, this disorder affects sometimes all the muscles of voluntary motion; in other instances it attacks those of one side only, just as the muscles of one side are affected, either in hemiplegia, or in some cases of epilepsy: hence it may be inferred that the morbid cause acts immediately on the common centre of the motive power, which is in the encephalon; lastly, in fatal cases of chorea, for it is sometimes fatal, there is found more or less of effusion on the surface or in the ventricles of the brain. But chorea, though its proximate cause, or the immediate cause of its phenomena, is an affection of the brain, is not discovered by experience to be most successfully treated by remedies applied to the head, such as are found to be useful in other cerebral affections. It appears that the primary cause is in some other part of the body, and the affection of the brain is but an intermediate link between it and the symptoms which manifest themselves. It is by removing the primary cause that we cure the disease. This is often an unhealthy condition of the alimentary canal, and chorea may be cured in many instances by purgative medicines. I have seen a case of chorea which had already induced extreme debility, and the appearance of imbecility, cured by a course of purgative medicines alone. This plan was first recommended by Dr. Hamilton, of Edinburgh, in a well-known work on purgative medicines. It was pursued in the case to which I am now referring: the only adjunct to purgatives was the shower-bath, and the patient began immediately to improve, and was soon completely well. I generally begin the treatment of a case of chorea by ordering a black draught every morning, and sometimes find this all that is required. The cases of chorea which I have found curable by purgative medicines alone have been those of boys: I know not whether this may be accidental, but I rather think not. In most instances, in females, the nervous system appears to be too much shaken, and the brain too much disordered, to be thus easily curable by purgatives unassisted, and that especially when the disease has been occasioned by a fright. In these cases metallic tonics are found to be the most useful auxiliaries. It has been the custom to use chiefly the oxide of zinc in this hospital: we find it the most efficacious as well as the most easily administered remedy of this class; it is given at first in small doses, as 5 or 6 grains, three times a day, which are gradually increased to large ones, such as 20 or 25 grains. In the course of 25 years I have seen but one or two cases in this Infirmary in which this remedy failed. It is worthy of note that this treatment seems never or scarcely ever to be successful in out-patients: the cause is obvious—a regular and simple diet, such as these patients have in the wards of the Infirmary, is a necessary condition for the cure.

We have in the Infirmary at present two cases of cerebral disease, which have been treated by issues on the scalp, made in one instance by incision, and in the other by the application of caustic. One of these cases is that of a young woman, named Shipway, in No. 2; and the other of a girl, named Kirkby, in No. 4. Mary Anne Shipway had been nearly twelve months in the Infirmary before the issue was ordered. She was admitted on account of some surgical complaint, and afterwards sent into the medical wards: there she laboured under some pain in the right side of the head under the parietal bone. Her disorder was treated by the ordinary remedies, not inactively, but without arresting its progress; it increased; the pain was most distressing, and without intermission: the seat of the disease was apparently the arachnoid, or the surface of the brain; her faculties became impaired: she was at length so demented or imbecile, that it was a question whether she should be sent to St. Peter's Hospital, where there are wards for insane persons and idiots. It seemed right, however, to make a trial of this remedy in the first instance, and a long issue was formed on the scalp over the sagittal suture, by means of the alkaline caustic, which was applied in the form of a paste. Some time elapsed before any improvement took place in the intellect of the patient, but she soon obtained a remission of pain; she was still 'silly,' as the nurse and the patients in the ward observed. However, she has now recovered an entire possession of her faculties, and she has scarcely any remains of the pain which formerly tormented her: her only suffering is for a short time after the issue has been dressed. There seems little room for doubt that we may soon venture to remove it. The other case, to which I have referred, is a patient of Dr. Wallis. I make no scruple of mentioning it, as I am sure he would not object to my so doing. This girl laboured under typhoid fever, affecting the brain. She sank into a state of complete coma; lay perfectly insensible, passing her evacuations involuntarily, and incapable of being roused. She appeared almost moribund. An incision was made over the sagittal suture, by Dr. Wallis's direction. The patient became soon more sensible, and is now convalescent.

This method of applying counter-irritation in diseases affecting the brain has been until lately nearly peculiar to the Bristol Infirmary. I wish particularly to call your attention to the fact that we never make use of it except in cases of the most severe and intractable disease; it is a severe remedy, though more so in appearance than in reality, and should only be used in cases in which all milder means have failed, or afford no expectation of benefit. I have been informed that in London this practice has been very much censured. It has been represented that we have recourse to it on trivial occasions. I have been told that a metropolitan teacher of medicine, when going through a hospital with his pupils, has been heard to address a patient, having some comparatively trifling affection, in such terms as these:—"It is lucky for you that you are not in the Bristol Infirmary, or you would have had your head cut open before now!" If any of you should chance to hear observations of this kind, you will, I am sure, correct such a mistake, and assure the party under so erroneous an impression that we never adopt the method which he censures, but in cases which admit of no other hopeful means, either of preserving life when about to be extinguished by disease of the brain, or restoring the intellectual faculties, or of curing amaurosis, or inveterate cases of epilepsy; and under such circumstances it has been found, under the blessing of divine Providence, to have been in some cases completely effectual.—*London Medical Gazette.*

---

*Causes of Sudden death—What is life?*—At a meeting of the Westminster Medical Society, October, 31, 1840, after some preliminary observations, Mr. Winslow stated that sudden death was of much more frequent occurrence than it used to be, and that therefore the subject was deserving of the serious consideration of the profession; and as medical men were always looked to in the course of justice to determine the cause of sudden death, it was not less their interest than duty to learn the exact state of knowledge respecting it. He considered that all diseases were invariably indicated in their early stages by certain signs, by which their existence might be predicated; and by paying attention to the incipient manifestations of latent organic disease, we might often prevent the development of those serious vital affections that suddenly extinguish life. After referring at some length to the physiological causes and phenomena of sudden death, he brought before the society an analysis he had made of 200 cases in which life was suddenly extinguished, from which it appeared that 40 arose from diseases of the heart; 20 from affections of the brain; 25, brain conjoined with disease of the heart; 18, abdominal affections; 20, aneurismal tumours; 10, convulsions; 32, mental excitement, with and without bodily disease; whilst under the use of mercury, 2; from lightning, 2; during parturition, 6; idiopathic asphyxia, 4; drinking cold water, 5; pulmonary apoplexy, 5; hæmorrhage from the Fallopian tubes, 1; air in vessels of brain, 2; blows on the stomach, 4; &c., &c. The majority of these cases were women. In 1838, Mr. Farr states that, out of 3012 cases of sudden death that occurred, 1840 were males and 1172 were females. Women, it appears, have less chance of dying suddenly than men, in the proportion of 10 to 18. The majority of cases of sudden death arise from hæmorrhage. From Jan. 11 to October 17, 1840, 524 instances of sudden death took place in the metropolis alone. Between the ages of 1 and 15, 142; between 15 and 60, 246; and from 60 upwards, 131. Mr. Winslow then considered in the following order the organic affections which commonly give rise to this awful calamity:—1, Diseases of the heart and large vessels; 2, Diseases of the lungs, and those causes that interfere with the functions of respiration; 3, Diseases of the brain and appendages; 4, Diseases of the stomach and abdominal viscera generally. The affections of the heart, that often predict a sudden suspension of life, are as follows:—Rupture of the heart; relo-condition of the heart, or asphyxia idiopathica; syncope angina pectoris; hydatids of the heart; metastasis of disease to the heart; the sudden bursting of aneurismal tumour. Mr. Winslow related many instances of fatal rupture of the heart. The first case is related by Harvey, and Morgagni, who himself died suddenly of this disease, narrates many similar cases. He considered that the symptoms which usually indicated liability to this affection are —violent pain beneath the sternum and in the arms, pain in the præcordia and epigastrium, cold extremities, &c. In the majority of cases the heart has undergone some previous disease, either ulceration or softening, or there is a disproportion in the thickness of its muscular parietes. When the heart has undergone a structural alteration, it requires but a slight cause to rupture it. Mr. Winslow referred, at considerable length, to sudden death from *asphyxia idiopathica*, a decisive point noticed by Mr. Chevalier, which consists of a sudden loss of power in the minute vessels to propel the blood they have received from the heart; in consequence of which this organ, after having contracted so as to empty itself, and the dilated organ, continues relaxed for the want of the return of its accustomed stimulus, and dies in that dilated state. This affection seizes the patient suddenly, and, if proper remedies be not administered, death ensues in a few moments. It is often mistaken for apoplexy. Mr. Winslow then alluded to sudden death from *protracted syncope*. A person drops down in a fainting fit; relief is not instantly afforded, the heart never recovers its action, and death ensues. A case of this kind was related, caused by wearing tight clothes. Mr. Winslow thought that death often resulted from *spasm of the heart*. Hippocrates, Herophilus, and Bichat, relate cases of this character. In this way the latter physiologist considered that great mental emotion caused death. Mr. Winslow entered into the consideration of sudden death resulting from the transmission of disease to the heart, to the bursting of aneurismal tumours, hydatids in the heart, vomica suddenly bursting in the substance of the lungs, effusion in the chest, ulmonary apoplexy, which disease was considered generally to be conjoined with disease of the heart. The affections of the brain that caused sudden death were next dwelt upon; viz., 1, Cerebral apoplexy; 2, Latent inflammation, causing suppuration; 3, Abscesses suddenly bursting; 4, Generation of air in the vessels of the brain. He considered that sufficient attention was not paid to the latent or insidious affections of the centre of the nervous system; that inflammation and the formation of pus often took place, and extinguished life before we were aware of its existence. Many cases, illustrative of this position, were related. Morgagni conceived that sudden death often resulted from what he termed "a repletion of the blood-vessels of the brain by air," which had been developed there spontaneously, compressing, by its rarefaction, the organ of the nerves, and thus destroying life. Mr. Winslow related the particulars of several instances of sudden death, in which the vessels of the brain were found filled with air; but expressed a doubt as to whether it was in our power to trace a connection between the extinction of life and the generation of this fluid in the vessels of the brain. The affections of the stomach and intestines were next considered. Many fatal cases of ulceration of the stomach, and intestinal canal were referred to, caused by the presence of *lumbrici*, in which death was suddenly and unexpectedly induced. The other affections which Mr. Winslow brought under the notice of the society, as deserving of its consideration, were as follows:—Death from mental excitment; drinking cold water; rupture of the biliary ducts; hæmorrhage from the Fallopian tubes in utero-uterine conception; inhalation of noxious gases, whilst under the influence of mercury, caused by exposure to intense cold, or to the heat of the sun.

Dr. Johnson considered that sudden death should be restricted to those cases in which death occurred instantly and mysteriously. He should not include apoplexy, when death was preceded by coma or other symptoms, among cases of sudden death. In his own experience he could only refer to three classes of cases of sudden death. The first consisted of cases of hydrothorax, the second of aneurisms, and the third of affections of the heart. He had found the first to be the most frequent among the causes of sudden death; although, generally, he believed that heart disease was the most frequent cause.

Dr. Leonard Stewart referred to cases of sudden death, arising from obscure diseases of the spinal marrow, and from dislocation of the vertebræ.

Mr. Wade related two cases of sudden death, arising from the presence of fibrinous concretions in the cavity of the heart, and which caused death by blocking up the auriculo-ventricular opening. In one case the patient suffered much from palpitation, and was bled by her medical attendant to the extent of three or four ounces. This produced syncope, from which she did not recover. There had been no previous symptoms, except occasional attacks of dyspnœa, with cough. She got up well on the morning of her death, and was seized with the palpitation of the heart, and great distress in breathing, suddenly. In the other case, the patient had been the subject of puerperal convulsions, which she apparently recovered from under the employment of large blood-lettings. She fell back, however, having been seized with palpitations, and died suddenly.

Dr. Marshall Hall thought that Dr. Johnson had restricted the subject of sudden death within too narrows limits. Sudden death might be forseen or unforseen. The former was the case in detected disease of the heart and large vessels; the latter he had known to occur in the state of exhaustion from loss of blood, and in the bloodless condition which obtains in chlorosis. One lady, after uterine hæmorrhage was going on well, rose from bed in order to evacuate the bladder, and sunk down and expired immediately. He had known three instances of sudden death from chlorosis, under similiar circumstances of effort and the upright position. Sudden death occurred in some cases of colica pictonum: this subject was mentioned by Heberden, Louis and Andral. It also occurred when the system was under the influence of mercury, with or without previous symptoms of erethismus mercurialis. It was well known that sudden death occured in angina pectoris. When there was ossification of the coronary arteries, the cardiac circulation or that within the substances of the heart itself, was probably interrupted, and the motion and the vitality of this most vital organ were extinguished at once. Dr. Hall had met with an interesting case, in which the usual symptoms of angina pectoris were present; and sudden death, which had been predicated, occurred In this case the heart was found to be loaded with fat. This he considered had arrested the cardiac circulation mechanically. The coronary arteries were free from disease. He thought that other diseases of the heart, as hypertrophy, valvular disease, &c., might induce sudden death, by arresting the circulation in the substance of the heart itself. He did not agree with Mr. Winslow in his enumeration, after Bichat, of the relative importance of the vital organs, viz.: the brain, the lungs, the heart. The brain was not so necessary to life as Bichat had supposed. Not only did a tortoise, the brain of which had been removed by Redi, live many months; and the wound, in the salamander decapitated by Dumeril, actually heal; but the fowl, from which M. Flourens removed the cerebrum, survived a very considerable time. It was true that the encephalous human fœtus was not *viable*, but it might live for fifty hours; and very young animals bore the loss of the cerebrum in the inverse ratio of their age. Neither was the brain, as stated by Mr. Winslow, the source of respiration. It was the medulla oblongata, according to the discovery of Legallois, which was instantly and absolutely necessary to the performance of this function. A patient died suddenly in the water-closet; an event by no means rare, and worthy of attention. On examination, a clot of blood was found compressing the medulla oblongata. Death, no doubt, had taken place instantaneously. Dr. Hall defined life, to be "the result of the pressure of arterial blood within the vascular structure of the different organs." If this pressure, or the arterial character of the blood were deficient, death ensued; if, therefore, the heart, or the lungs, ceased to act, if the blood itself failed suddenly, sudden death occurred. The medulla oblongata (not the brain,) the lungs, and the heart, were, as had been expressed, the "tripod of life:" and was not the blood borne on this tripod? Life then, in a word, was arterial blood. If any part of this tripod, or the blood it supported, failed, sudden death occurred. This was true at least in animals high in the scale. Reptiles bore the loss of the heart without immediate death, perhaps without the immediate loss of sensation: the pressure and counter-pressure between the blood and the organs must be carefully maintained. If the pressure were augmented in an organ, we had apoplexy of that organ; if diminished, syncope, if so free a use of those terms might be allowed. If these morbid states took place in an extreme degree, we had death; if the arterial quality of the blood were deficient, we had death from this cause.

Such seemed to be the physiology or pathology of death; to such conditions must, he believed, all cases of sudden death be eventually traced. Before concluding, Dr. Hall referred to another case of sudden death. When a patient had laboured under obstructed bowels, when the obstruction had been overcome, and when all was hope, he had observed the patient suddenly and unexpectedly to sink. In the case of a little boy, whom he had attended, intus-susception had actually been overcome, the bowels had acted but the powers of life sunk. On a post-mortem examination, the intus-suscepted portion of intestine was found swollen, and of a deep red hue, but without any actual destruction of its texture. A similar event was known to have occurred after the reduction of, or the operation for, strangulated hernia.

Mr. Snow, said, that death, in the ordinary acceptation of the term, consisted in the stoppage of the respiration and circulation, and was consequently merely a state of suspended animation, as there was a molecular or vegetative life which continued for some time afterwards. Syncope seemed to commence sometimes in the heart and sometimes in the nervous centres. Whilst bleeding a patient freely, there was sometimes no alteration in the pulse until the moment the patient fainted; at other times, the heart acted more and more feebly from the loss of its natural stimulus, until, probably, the blood was not sent to the brain with sufficient force to maintain its functions, and suddenly an influence was communicated to the heart which stopped its action. Extensive loss of blood might cause death at once; and sometimes, when not immediately fatal, sudden death would take place perhaps on change of posture. The contra-stimulant medicines produced similar effects; for instance, digitalis, belladonna, lead, oxalic acid, &c., might, in a large dose, cause death at once, or by repeated smaller doses the action of the heart might be so lowered, as to render a patient liable to sudden and fatal syncope. Sudden death took place occasionally in scurvy, chlorosis, and other diseases of great debility. Malaria and the poison of contagious diseases, which were likewise sedative or contra-stimulant agents, sometimes caused sudden death without developing their peculiar effects. After these considerations there could be no doubt but sudden death might take place when there was no organic disease; and he related a case in which no alteration of structure was found. He could not agree with the author of the paper, that in asphyxia the action of the heart was stopped through the medium of the brain; true, it was mechanically stopped by the accumulation of blood which could not passs through the lungs, but it retained its contractility; and hence the occasional success of artificial respiration after sensibility was extinct; for when those chemical changes were again set up in the lungs which enabled the blood to pass, the heart being relieved of its load, again began to act. He thought that artificial respiration ought to be tried in children just dead from convulsion,—in cases of narcotic poisoning,—and, in fact, all cases of asphyxia, where there was not disease that was incompatible with life; and he considered that the proper method of instituting artificial respiration, was that which had been laid before the society by Mr. Read, namely, to exhaust the lungs, when, by the mechanical resiliency of the ribs and respiratory muscles, the lungs became refilled by the atmospheric pressure, in the manner of natural respiration.

Dr. G. Bird referred to the cases of sudden death dependent upon air in the vessels of the brain, as related by the author of the paper, on the authority of Morgagni, and stated, that in the three cases detailed by the eminent pathologist, as those in which he supposed this phenomenon had occurred, there were ample sources of fallacy. In all of the cases the body was more or less advanced in putrefaction. In the first case, in which an Ethiopian had died whilst blowing a trumpet, there was serous effusion in the ventricles, and fluid at the base of the brain. In the second instance, the patient, a fisherman, had a gangrenous hernial cyst; and the third patient had a large flabby heart with an adherent pericardium. Here, then, in all the cases there was sufficient disease to account for death, independent of air in the vessels of the brain.

Dr. Guy referred to the case of sudden death which occurred in the house of Dr. Turnbull, whilst a boy was having air injected into the ear. In this case there was blood effused on the surface of the brain, between the pia mater and the dura mater. It appeared difficult to decide in this case, whether death resulted from the operation which the patient was undergoing, or whether it was dependent upon the great muscular exertion which he had taken a few minutes before sitting down. It might in this instance have arisen from both causes. Some of the difficulty, however, would be cleared away, if it could be decided whether or no the boy could have existed half an hour without symptoms, with such an extravasation of blood as had been mentioned. It appeared on evidence that the pressure of air into his ear, on several previous occasions, had been attended with no bad results.

Mr. W. Elliott related three cases of sudden death from a mental shock, in which no structural disease could be detected. In one instance the patient was a strong athletic woman, thirty-five years of age. In the second case, the patient died on the night-stool. She had suffered some time from jaundice; a large gall-stone was found impacted in the ductus choledochus. In the third case, the patient died from the shock produced by the passage of a catheter into the bladder; he fainted, and did not recover.

Mr. Brunsgill, thought it was of the greatest importance to first define in what life consisted. He thought it probable that electricity had much to do with the matter, and considered that the respiratory apparatus might be considered to give off positive, and the stomach negative, electricity.

Dr. Reid referred to sudden deaths occurring on the operating table, and mentioned a case of the kind which happened to Dupuytren. This eminent surgeon was removing a small tumour from the scapula of a young woman, when she fell back and expired. Death was attributed to the ingress of air into the circulation, stopping the heart's action. Mr. John Bell had detailed a similar case. Dr. Reid doubted whether this explanation of the mode of death was correct.

Mr. Gregory Smith detailed a case of sudden death in an infant four days old, arising apparently from the pressure of the bandage usually applied to young children on the chest, and the distention of the stomach from recent sucking. There was no sign of any convulsion having occurred.

Dr. A. T. Thomson detailed a case in which animation was suspended in a young lady of seventeen years of age, from tight lacing.

She was sitting at dinner, and had just taken some soup, when she fell back lifeless. She was restored by cutting open the stays, which separated with a very loud noise. Dr. Thomson referred to the valuable series of reports which were issued from the General Registry-office, and thought that the great number of sudden deaths registered as occurring from apoplexy, had arisen from errors in those persons who delivered the reports to the various registrars. He thought this subject did not receive due investigation.

Dr. Stewart related some cases of sudden death, depending entirely on change of posture, in persons who had been long bed-ridden.

Mr. Wade had seen several cases of sudden death from the impaction of a foreign body in the trachea.

Mr. Streeter had seen young children die suddenly in bed from spasm of the glottis. He could not understand how in Mr. Smith's case death was produced, except from this cause. He had seen cases similar to that related by Dr. Thomson. Removing the busk of the stays generally afforded some relief.

Dr. Chowne made some remarks on the difficulties which were thrown in the way of arriving at the exact cause of sudden death, by the coroner's court. In cases even where post-mortem examinations were ordered, they were generally at so late a period as to render the case obscure. He thought that our knowledge of the causes of sudden death, and medical science generally, was obstructed by this mode of proceeding.

Mr. Winslow rose at a late hour to reply; he briefly recapitulated the observations which had been made on his paper, and referred to the remarks of Dr. Thomson, in regard to the errors which had occurred in registering the causes of sudden death. It was probable that many cases, supposed to be apoplexy, were not really so. Out of 22,452 cases of sudden death occurring in Milan, only 875 were from apoplexy. The majority of these were males, and during the winter months. Mr. Winslow then related some cases of sudden death from drinking soda-water, from the spontaneous generation of gases in the blood, and from gluttony. He agreed in thinking with Mr. Farr, in his admirable letter to the Registrar-general, that many sudden deaths occurred from the employment of concentrated poisons, which are attributed to other causes.—*Lancet.*

---

*Hepatic Abscess*; *Discharge of a Biliary Calculus.*—Mad. N., 73 years of age, presented the external signs of an organic affection of the liver. The pain in the right hypochondrium became excessively severe, and high febrile symptoms came on.

After a few days, a phlegmonous swelling made its appearance at the seat of the pain; this quickly assumed all the signs of an abscess, so as to induce the surgeon in attendance to make an opening into it. A large quantity of purulent matter mixed with bile and blood flowed out, and to prevent the healing of the wound, a tent was introduced and left in it. After the lapse of a few days, on introducing a probe along the fistula, a foreign body was felt distinctly. The wound being enlarged, this was extracted and proved to be a biliary calculus of nearly three inches and a half in diameter.—*Med. Chir. Rev.*

---

*Mr. Donovan on the Hydrocyanoferrate of Quinina.*—This salt has been brought forward by Signior Bertozzi, of Cremona, as a substitute for sulphate of quinina, where that fails. Doctors Zaccarelli and Carioli have confirmed his statements and anticipations. Mr. Donovan gives directions for procuring the salt, for which we must refer to our contemporary.

The hydrocyanoferrate of quinina, when in small fragments, is of a pea-green colour; its taste is intensely bitter; it dissolves in cold, but better in hot alcohol, and is precipitated almost entirely from the solution by water. In prescription, it would be an error to promote its solution in water by means of dilute sulphuric acid, as is done in the case of sulphate of quinina; the salt would be decomposed by this acid, and the solution would become blue. It ought not to be prescribed with tincture of cinchona, and consequently not with infusion or decoction. The dose given by Dr. Zaccarelli, was equal to three grains and a half troy, repeated according to necessity.

Although this febrifuge is precipitated by water from its alcoholic solutions, it separates in the state of so fine a powder, and remains so long suspended, that it will answer for exhibition very well in this state. The following formula will be found convenient:

℞. Hydrocyanoferratis quininæ grana quatuor,
Spiritûs rectificati drachmam. Solve.
Adde aquæ, vel
Misturæ camphoratæ drachmas septum.
Misce fiat haustus, ut res nata sit, phialâ prius agitatâ, sumendus.

In pills

℞. Hydrocyanoferratis quininæ grana viginti quatuor,
Mucilaginis gummi Arabici, q. s.
fiat massa quam divide in pilulas duodecim.

These pills will be of a proper size, and two of them will constitute a dose; to be repeated according to the discretion of the prescriber.

Mr. Donovan thinks the liquid form the better. He recommends, and so do we, the medicine to the profession.—*Dublin Journal.*

